# Fabrication of Cu_2_ZnSnS_4_ (CZTS) Nanoparticle Inks for Growth of CZTS Films for Solar Cells

**DOI:** 10.3390/nano9030336

**Published:** 2019-03-02

**Authors:** Xianfeng Zhang, Engang Fu, Yuehui Wang, Cheng Zhang

**Affiliations:** 1 Zhongshan Institute, University of Electronic Science and Technology of China, Zhongshan 528402, Guangdong, China; wangzsedu@126.com (Y.W.); aqian2006@gmail.com (C.Z.); 2 State Key Laboratory of Nuclear Physics and Technology, School of Physics, Peking University, Beijing 100871, China; efu@pku.edu.cn

**Keywords:** Cu_2_ZnSnS_4_ solar cell, ball milling, nano-ink, annealing

## Abstract

Cu_2_ZnSnS_4_ (CZTS) is a promising candidate material for photovoltaic applications; hence, ecofriendly methods are required to fabricate CZTS films. In this work, we fabricated CZTS nanocrystal inks by a wet ball milling method, with the use of only nontoxic solvents, followed by filtration. We performed centrifugation to screen the as-milled CZTS and obtain nanocrystals. The distribution of CZTS nanoparticles during centrifugation was examined and nanocrystal inks were obtained after the final centrifugal treatment. The as-fabricated CZTS nanocrystal inks were used to deposit CZTS precursors with precisely controlled CZTS films by a spin-coating method followed by a rapid high pressure sulfur annealing method. Both the grain growth and crystallinity of the CZTS films were promoted and the composition was adjusted from S poor to S-rich by the annealing. XRD and Raman characterization showed no secondary phases in the annealed film, the absence of the detrimental phases. A solar cell efficiency of 6.2% (open circuit voltage: *V*_oc_ = 633.3 mV, short circuit current: *J*_sc_ = 17.6 mA/cm^2^, and fill factor: FF = 55.8%) with an area of 0.2 cm^2^ was achieved based on the annealed CZTS film as the absorber layer.

## 1. Introduction

In recent years, kesterite Cu_2_ZnSnS_4_ (CZTS) and Cu_2_ZnSn(S, Se)_4_ (CZTSSe) solar cells have drawn attention because of their promise as an absorbing layer for applications in thin-film photovoltaics owing to its low cost, nontoxicity and earth abundance of its elemental components as well as an adjustable bandgap [1,2,3]. One advantage of CZTS over other kinds of chalcopyrite-related solar cells is its suitability for achieving high efficiency solar cells through nonvacuum fabrication methods. Furthermore, the world record conversion efficiency of CZTSSe solar cells is currently 12.6% [4] based on a hydrazine pure solution approach. There have been several reports on the fabrication of CZTS or CZTSSe solar cells. Both vacuum methods, such as sputtering [5], coevaporation [6], epitaxial methods [7], and nonvacuum methods [8,9,10,11], have been reported. Nonvacuum methods are lower in cost and more suitable for mass production than are vacuum methods. Among nonvacuum methods, the highest conversion efficiency CZTS solar cells are based on molecular precursor solutions or nanoparticle dispersions [12,13]. Although these kinds of fabrication methods are appealing because of their low complexity, low-cost, and scalability, such methods are complicated by the need for toxic solvents or metal–organic solutions that contain large amounts of organic contaminants, which induce cracking during the following annealing process [14,15]. The use of the toxic and unstable solvent hydrazine requires all processes for ink and film preparation to be performed under an inert atmosphere. As a result, it is difficult to adapt this approach to low-cost and large-scale solar cell fabrication. 

In this work, we report a simple technique for fabricating CZTS nanoparticles ink by a wet ball milling method using nontoxic ethanol and 2-(2-ethoxyethoxy) ethanol as the solvents. A similar study has been reported by Woo, et al.; an efficiency of 7% was achieved [16]. However, the use of CZTS powder to fabricate CZTS film is expected to have the following benefits. (1) The fabrication process is simpler. (2) The growth of grains will be promoted because the grain boundary meltdown temperature is lowered. (3) Stoichiometric film compositions are easier to obtain because the chemical reactions are less complicated. The use of nontoxic solvents is more cost-effective and environment friendly, which is important for practical photovoltaic applications. The ink was used to fabricate CZTS thin films (precursors) by a spin-coating method, followed by annealing the precursor in a sulfur-rich atmosphere. The commercial CZTS powder was obtained from Mitsui Kinzoku, and detailed information of its characteristics is currently unavailable. The sulfur vapor not only prevents the formation of volatile Sn–S compounds but also supplies S atoms to make the CZTS films sulfur-rich, which is a requirement for high performance solar cells. The procedure for fabricating CZTS films from CZTS powder is reported in detail in this paper.

## 2. Experiment Details 

### 2.1. Sample Preparation 

**Figure 1:**
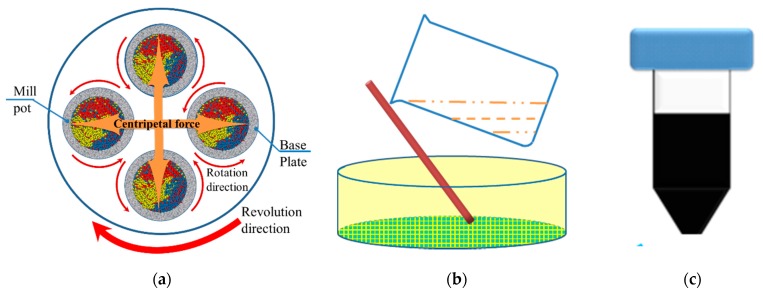
Fabrication process of Cu_2_ZnSnS_4_ (CZTS) nanoparticle ink: (**a**) schematic of ball milling machine, (**b**) filtration of CZTS particles smaller than 32 μm, (**c**) nanoparticle ink of CZTS.

Figure 1a–c illustrates the process for fabricating CZTS nanoparticle ink. In the ball milling system, a 1-mm ball, 50-μm ceramic balls, and CZTS powder were mixed together in the mill pot. A 5-mL portion of ethanol was added to improve the wet milling effect. Figure 1a shows a schematic diagram of the milling system. The milling pots were rotated along their own axis together with the base plate. The milling process was performed for 40 h. After ball milling, the whole mixture was strained through a filter screen to obtain particles smaller than 32 μm, and nontoxic ethanol and 2-(2-ethoxyethoxy) ethanol were used to wash the milling ball to increase nanoparticle recovery, as shown in Figure 1b. Through this procedure, the milling balls and large particles of CZTS (>32 μm) were removed whereas a mixture of relatively small CZTS particles (<32 μm) and the solvents were retained. We used 2-(2-ethoxyethoxy) ethanol as a dispersion agent to prevent coagulation of the nanoparticles, and ethanol was used to reduce the viscosity of the solvent and promote precipitation of large particles during the following centrifugation. The resulting solution was then ultrasonically processed to disperse the particles in the solvents for 1 h. 

**Figure 2:**
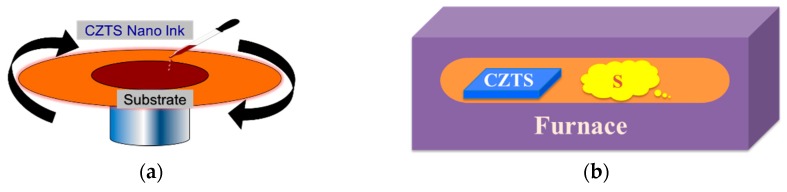
Fabrication process of CZTS films: (**a**) fabrication of CZTS precursor by spin-coating and (**b**) the sulfur vapor annealing process.

The as-prepared mixture of CZTS and solvents was first centrifuged at a low speed 1500 rpm to remove particles over several μm in size. The precipitate was disposed of and the upper layer of the solution was decanted for further centrifugal treatment. The aforementioned processes were repeated three times at a higher speed of 6000 rpm and the nanoparticles were obtained. The nanoparticle ink was obtained with a concentration of 200 mg/mL by adjusting the quantity of ethanol. The nanoparticle ink was then used to fabricate CZTS precursors by spin-coating. Figure 2a shows a schematic diagram of the spin-coating system. The substrate was rotated at a speed of 2000 rpm and the CZTS ink was dripped on at a speed of 5 μL/min. The final CZTS precursor film showed a thickness of 1–1.5 μm. Finally, the precursors were annealed in a sulfur-rich atmosphere to improve the grain size and crystallinity. The sulfurization process was conducted by sealing the precursor and powdered sulfur into a vacuum quartz tube with a length of 15 cm, which was placed in the annealing furnace (FP410, Yamato Company, Tokyo, Japan), as shown in Figure 1b. The furnace was heated to 600 °C within 15 min and the vapor pressure of sulfur was approximately 0.1 atm. The annealing process was performed for 20 min after the system achieved 600 °C. Then the sample was allowed to cool to room temperature naturally. 

**Figure 3:**
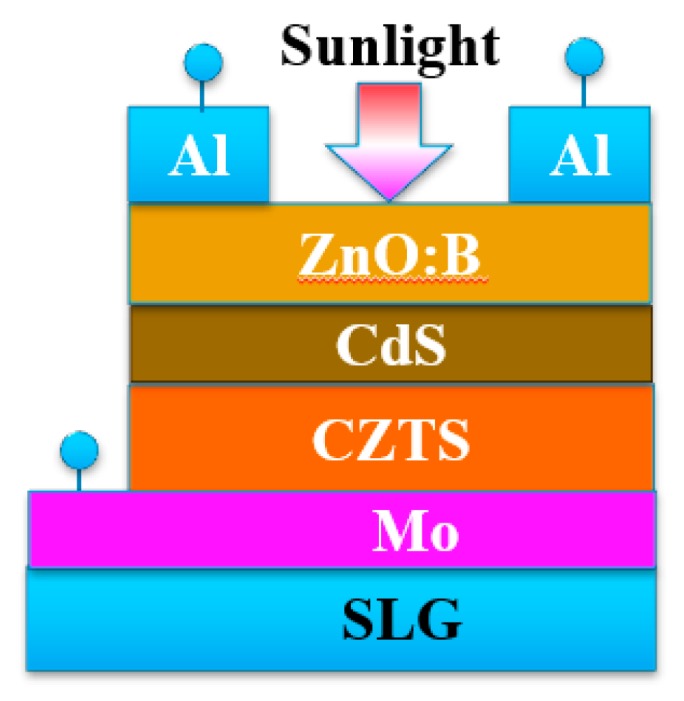
Structure of CZTS solar cell.

A typical structure of a CZTS solar cell is shown in Figure 3. The as-grown CZTS film was used as the absorbing layer. A CdS layer with a thickness of 50 nm was fabricated by a chemical bath deposition method as the buffer layer. Intrinsic ZnO with a thickness of 100 nm and B-doped ZnO with a thickness of 400 nm were then sputtered as the window layer. To measure the performance of the solar cell, an Al grid was evaporated as the front electrode. 

### 2.2. Characterization

The morphology of the annealed CZTS films was characterized with a scanning electron microscope (SEM, JSM-7001F, Tokyo, Japan) equipped with a JED-2300T energy dispersive spectroscopy (EDS) system (Tokyo, Japan) operating at an acceleration voltage of 10 kV. EDS, for compositional analysis, was measured at an acceleration voltage of 15 kV. The grain size distribution was measured with a transmission electron microscope (TEM, JEOL JEM-2100F, Tokyo, Japan). X-ray diffraction (XRD) analysis was performed with a Rigaku SmartLab2 with a Cu-K source and the generator was set to 20 mA and 40 kV. Raman measurements were performed with a RENISHAW-produced inVia RefleX type Raman spectrometer equipped with an Olympus microscope with a 1000 magnification lens at room temperature. The excitation laser line was 532 nm. The solar cell performance was measured with a 913 CV type current–voltage (J–V) tester (AM1.5) provided by a EKO (LP-50B, Tokyo, Japan) solar simulator. The simulator was calibrated with a standard GaAs solar cell to obtain the standard illumination density (100 mW/cm^2^). 

## 3. Results and Discussion

### 3.1. Centrifugation to Obtain CZTS Nanoparticle Ink

**Figure 4:**
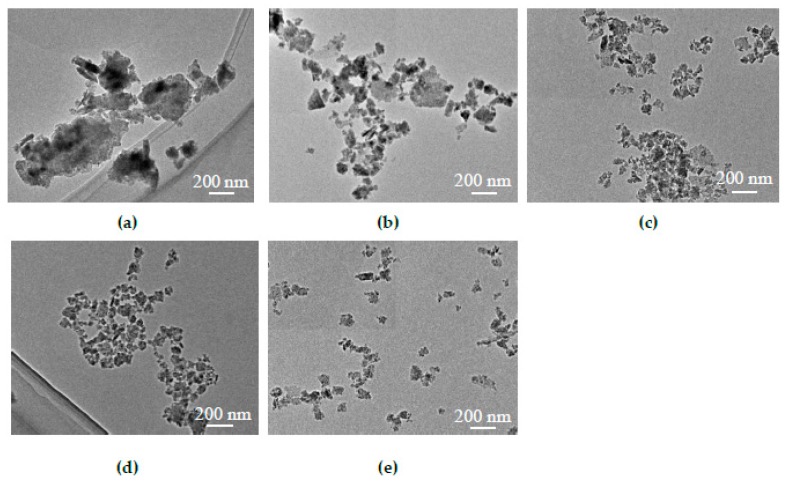
TEM images of CZTS dispersions with different centrifugal conditions. Distribution of CZTS particle size in inks with different centrifugal conditions (**a**) Without centrifugation; (**b**) 1500 rpm for 10 min; (**c**) 6000 rpm for 10 min; (**d**) 6000 rpm for 20 min; and (**e**) 6000 rpm for 30 min.

Figure 4a–e shows TEM images of the CZTS particle distribution of the dispersion subjected to different centrifugation conditions. Figure 4a shows the distribution of CZTS particles for the CZTS dispersion without a centrifugal treatment. The small particles and large particles agglomerated together to form large clusters such that the boundaries between particles became unclear and it was not possible to tell the size of the particles; hence, the larger and smaller particles and nanoparticles were not separated. Figure 4b shows an TEM image of the CZTS ink centrifuged for 10 min at 1500 rpm. A portion of the large particles was removed, which reduced the agglomeration. The particle boundaries were clear; however, particles larger than several hundred nm remained. To further reduce the size of the particles, the dispersion was centrifuged at a high speed of 6000 rpm for 10, 20, and 30 min. The results are shown in Figure 4c–e, respectively. The sample shown in Figure 4c, had the largest particles (in the range of 100 to 200 nm) and almost no agglomeration was observed. In sample (d), particles remaining in the dispersion were smaller than 100 nm, indicating that nanoparticles were obtained. The particle size of sample (e) was in the range of 50 to 100 nm, which indicated that after the treatment to obtain sample (d), the particle size of the dispersion was no longer affected by centrifugation because of the limitations of final particle sizes generated by ball milling processes. 

### 3.2. Deposition of CZTS Precursors

**Figure 5:**
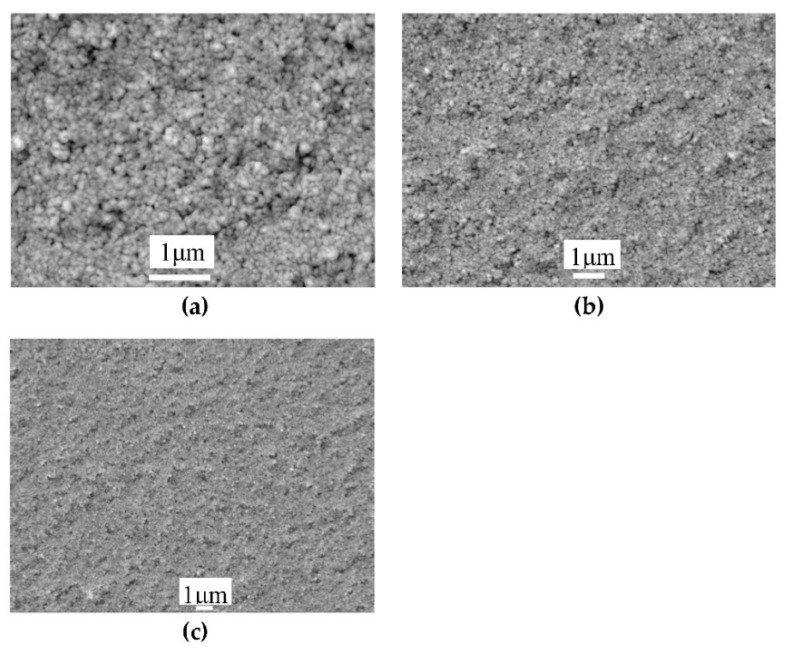
SEM images of a CZTS film deposited from the as-fabricated CZTS nanoparticle inks under different magnifications: (**a**) 20000×; (**b**) 10000×; and (**c**) 5000×.

The CZTS nanoparticle inks were used to deposit the CZTS precursors on glass substrates by a spin-coating method. The speed of the substrate was approximately 2000 rpm and 5 μL of CZTS ink was dripped at the center of the substrate for each drop, which was repeated 10 times to obtain a film with a thickness of 1–1.5 μm. Figure 5a–c shows the surface morphology of the CZTS film with different magnifications. The SEM image showed a compact morphology with grains smaller than 100 nm without cracks and no large particles were observed. The specific grain size could not be measured because of the small boundaries between grains. Because the precursor was only grown at room temperature, an additional high-temperature treatment was necessary to improve the grain size and crystallinity of the film. 

### 3.3. Annealing of the Precursor 

**Figure 6:**
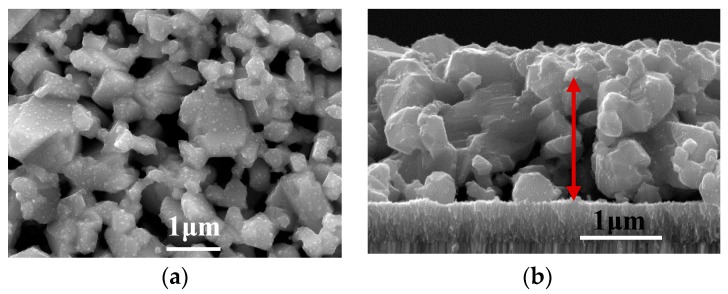
(**a**) Surface and (**b**) cross-section of an annealed CZTS film annealed at 600 °C in a S-rich atmosphere.

To induce grain growth and reduce the residual organic impurities, the CZTS precursor was annealed in an atmosphere with a high sulfur vapor pressure for 20 min at a temperature of 600 °C. Figure 6a,b shows the surface and cross-sectional SEM images of the CZTS films after annealing, respectively. Comparing the precursor morphology, as shown in Figure 5, the grain size increased markedly. The final grain size ranged from several hundred nm to several μm and cracks begin to appear between the grains, either because of grain growth or decomposition of the CZTS particles. According to the cross-sectional image (Figure 6b), the grains extended throughout the film in the thickness direction, which is expected for high-quality films. However, cracks stretching from the surface to the bottom of the film were also observed (marked by the red arrow), indicating the low density of the film. One explanation for this cracking was reported by Scragg, et al. owing to decomposition of CZTS film, as shown in following reactions (1) and (2) [17].

(1) $${\mathrm{Cu}}_{2}{\mathrm{ZnSnS}}_{4}⇌{\mathrm{Cu}}_{2}S\left(s\right)+\mathrm{ZnS}\left(s\right)+\mathrm{SnS}\left(s\right)+1/2S_{2}\left(g\right)$$

(2) SnS(s)⇌SnS(g)

One solution to overcome this issue is to reduce the annealing temperature to prevent equilibrium (1) from shifting to the right and extending the annealing time to ensure maintain the crystallinity. 

To make a comparison, CZTS film using centrifugation condition: 1500 rmp for 10 min was also annealed with the same annealing condition and completed solar cell structure (Please refer to the Supplementary Materials).

Table 1 shows the composition of the CZTS precursor and annealed film, as determined by energy-dispersive X-ray spectroscopy (EDX). The precursor had a sulfur composition less than 50% whereas the sulfur content increased to 50.5% after annealing, indicating that the film was converted from sulfur poor to sulfur-rich, which produces p-type CZTS films. It has been widely reported that Zn-rich (Zn/Sn > 1.0) films are required for fabricating high-performance CZTS solar cells [18,19], meaning that the composition of our CZTS films needed to be adjusted. One possible way to adjust the film to Zn-rich is to fabricate a thin layer of ZnS nanoparticles between the CZTS precursor and Mo back-contact, such that in the following annealing step, both Zn and S will be supplemented. 

**Figure 7:**
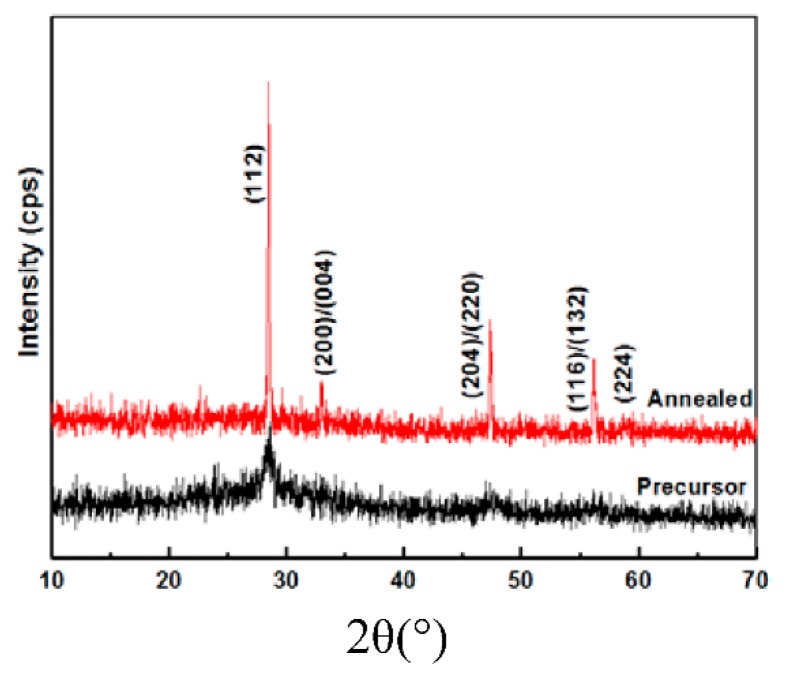
XRD of precursor and annealed CZTS film.

**Figure 8:**
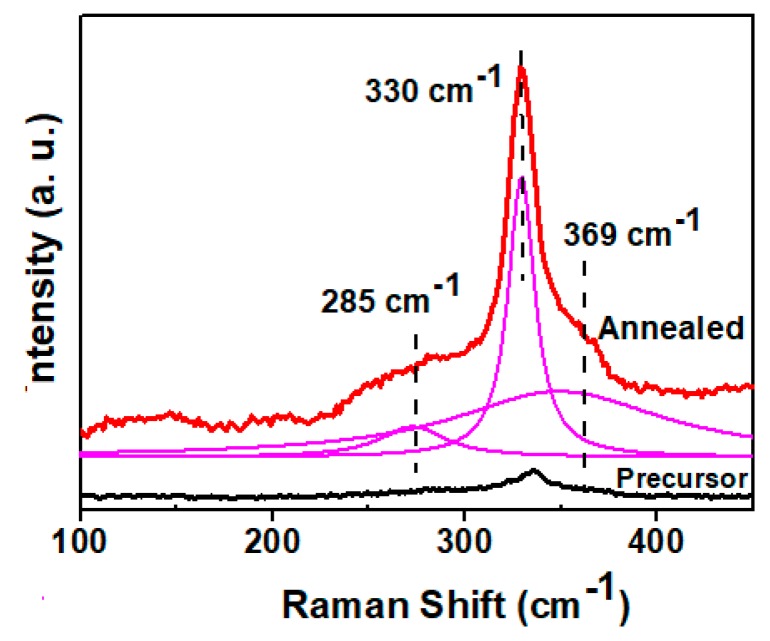
Raman spectrums of precursor and annealed CZTS film with fitting of the peaks using Lorentzian curve.

Figure 7 shows the XRD patterns of the precursor and annealed film of CZTS. The crystallinity was also improved by high-temperature annealing. The sulfurization process induced sharpening and strengthening of the peaks. All the peaks of the precursor and the annealed film were assigned to kesterite CZTS. No peaks of secondary phases, such as ZnS and Cu_2_S, which easily form at high temperatures [20], were detected by XRD. However, XRD alone is incapable of identifying small amounts of secondary phases because of its detection limits. To complement this method, we also performed Raman measurements to confirm the absence of secondary phases. Raman spectra of the precursor and annealed CZTS thin films are shown in Figure 8. The lower spectrum shows the annealed CZTS film with peak fitting by a Lorentzian curve. According to the figure, the precursor showed one peak at 330 cm^−1^, corresponding to the A mode of kesterite CZTS. The annealed film exhibited a typical Raman spectrum of kesterite CZTS films with three peaks at 285, 330, and 369 cm^−1^, corresponding to the two A symmetry modes and a B symmetry mode of the CZTS kesterite structure, respectively [21,22]. This result also indicated that no secondary phases are observed after the annealing process. 

The annealed CZTS films were used to fabricate complete solar cell structures. Solar cell performance was evaluated under standard conditions. The conversion efficiency of three cells on the same sample was measured as shown in Table 2. The solar cell ranged from 2.5% to 6.2%, indicating ununiform solar cell performance due to the poor film quality as shown in Figure 7. 

**Figure 9:**
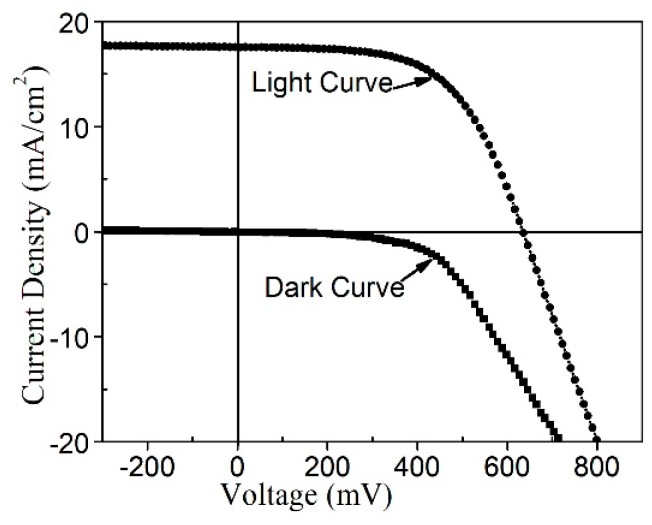
J–V curve of CZTS.

Figure 9 shows dark and light I–V curves of solar cell using annealed CZTS film as the absorber layer with best solar cell performance. The photovoltaic device exhibited an efficiency of 6.2%, with *V_oc_* = 633.3 mV, *J_sc_* = 17.6 mA/cm^2^, and FF = 55.8%, for an area of 0.20 cm^2^.

**Figure 10:**
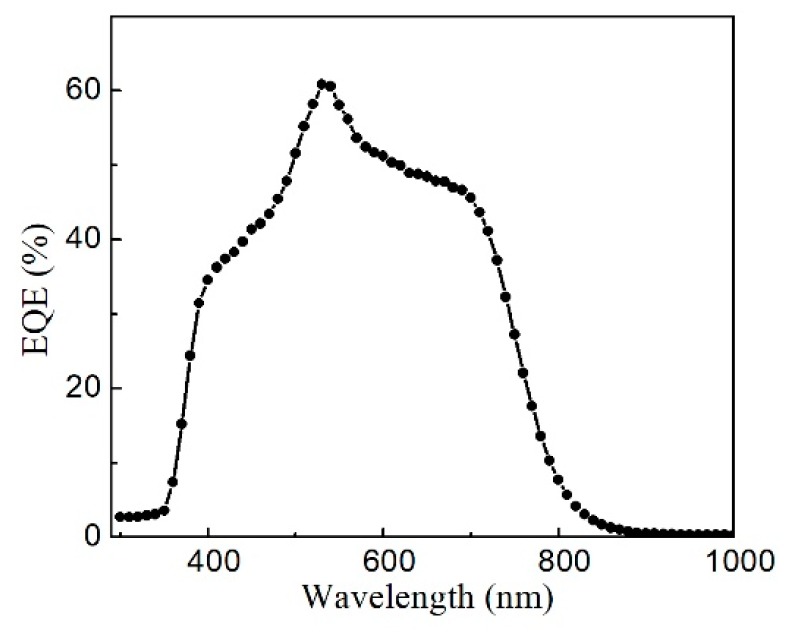
External quantum efficiency (EQE) of CZTS solar cell.

Figure 10 shows the external quantum efficiency (EQE) curve of the CZTS solar cell. Over the visible range of the solar spectrum, the maximum QE was less than 60%, indicating strong recombination. The QE curve decreased sharply in the infrared region at 770 nm, which is the CZTS absorption edge. Thus, the calculated bandgap of the CZTS films was approximately 1.61 eV. The features near 510 nm and 380 nm correspond to the absorption edges of the CdS and ZnO layers [23,24], which are commonly used CdS buffer and ZnO window layers. 

On the basis of the EQE data of a solar cell, *J_sc_* was calculated as [25]

(3) $$J_{sc}=q\int_{0}^{\infty }QE\left(E\right)b_{s}\left(E,\;T_{s}\right)dE$$

where, *q* is the elementary charge, *QE* is the quantum efficiency, and *b_s_* is solar flux or irradiation. For an air mass of 1.5, the data is available from Ref. [26]. On the basis of Equation (3), Figure 10, and the solar irradiation spectrum, *J_sc_* of the CZTS solar cells was calculated to be 14.2 mA/cm^2^, because the J-V curve represents the real performance of a photovoltaic device. The slight deviation of *J*_sc_ calculated from the QE curve can be explained by the fact that the QE measurement is performed at a single wavelength with a much lower intensity than one-sun irradiation.

## 4. Conclusions

We synthesized a CZTS nanoparticle ink by a wet ball milling method together with centrifugation treatments based on only nontoxic solvents. The ink was then used to deposit CZTS precursor films by a spin-coating method, which led to extremely flat surfaces with high-uniformity. The precursor was annealed at a high temperature of 600 °C under a sulfur atmosphere and the grain size increased to approximately 1 μm from the original size of less than 100 nm. Both the composition and crystallinity of the CZTS film were markedly improved by annealing. The absence of secondary phase formation during the annealing process was confirmed by XRD and Raman analysis. A solar cell efficiency of 6.2% (*V*_oc_ = 633.3 mV, *J*_sc_ = 17.6 mA/cm^2^, and FF = 55.8%) with an area of 0.2 cm^2^ was achieved using annealed CZTS film as the light absorbing layer. To improve solar cell performance, it is necessary to increase grain size, improve crystallinity, and reduce defects in the film. Because the fabrication process of CZTS features a complex growth mechanism, the formation of secondary phases should be checked to confirm film quality, which directly affects solar cell performance.

## Figures and Tables

**Table 1:** Composition of precursor and annealed film as measured by energy-dispersive X-ray spectroscopy (EDX).

	Cu (%)	Zn (%)	Sn (%)	S (%)	Zn/Sn	Cu/(Zn + Sn)
**Precursor**	25.4	9.9	15.6	49.1	0.63	1.00
**Annealed CZTS film**	24.7	9.8	15.1	50.5	0.65	0.99

**Table 2:** Performance of CZTS solar cells.

Sample No.	E*_ff_*	*V_oc_* (mV)	*J_sc_* (mA/cm^2^)	FF (%)
1	6.2	633.3	17.6	55.8
2	4.3	578.2	15.3	48.6
3	2.5	497.1	12.2	41.2

## Supplementary Materials

The following are available online at https://www.mdpi.com/2079-4991/9/3/336/s1, Figure S1: (a) Surface and (b) cross-section of an annealed CZTS film using centrifugation condition: 6000 rpm for 20 min. (c) Surface Morphology of an annealed CZTS film fabricated with CZTS ink using centrifugation condition: 1500 rpm for 10 min. The annealing was conducted at a temperature of 600 °C in S rich atmosphere, Figure S2: J-V curve of CZTS solar cells for (a) centrifugation 6000 rpm for 20 min; (b) 1500 rpm for 10 min, Table S1: Solar cell performance of CZTS solar cells.
