# Supplementary material for: Fabrication of Cu2ZnSnS4 (CZTS) Nanoparticle Inks for Growth of CZTS Films for Solar Cells
Source: Nanomaterials (Basel). 2019 Mar 2;9(3):336. doi: 10.3390/nano9030336 (PMC6473955; doi:10.3390/nano9030336)
Supplement: Supplementary file 1 [file nanomaterials-09-00336-s001.pdf]

Supporting document

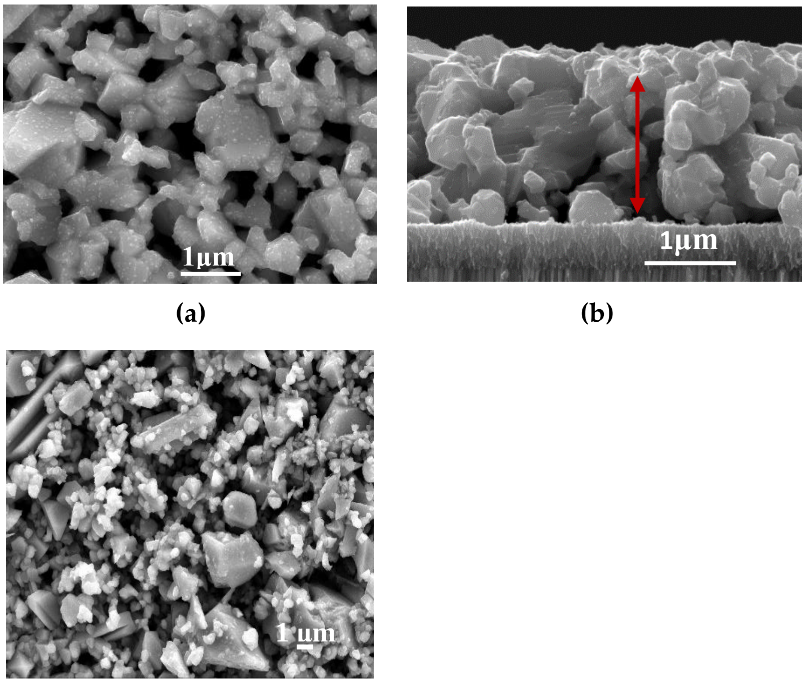

(c)

Figure S1 (a) Surface and (b) cross-section of an annealed CZTS film using centrifugation condition: 6000 rpm for 20 min. (c) Surface Morphology of an annealed CZTS film fabricated with CZTS ink using centrifugation condition: 1500 rpm for 10 min. The annealing was conducted at a temperature of 600 °C in S rich atmosphere.

Figure S1 (a) and (c) show morphology of annealed CZTS films with different centrifugation conditions. It is obvious that the film quality is affect by the particle size in the precursor. As explained in the main text, the grain size ranged from several hundred nm to μm level and cracks begin to appear between the grains when the centrifugation condition was 6000 rpm for 20 min. However, when the centrifugation condition was 1500rpm for 10 min, the quality of the annealed film was very poor and the grain size is nonuniform at all (the largest can go sever μm and the smallest is nano meter level).

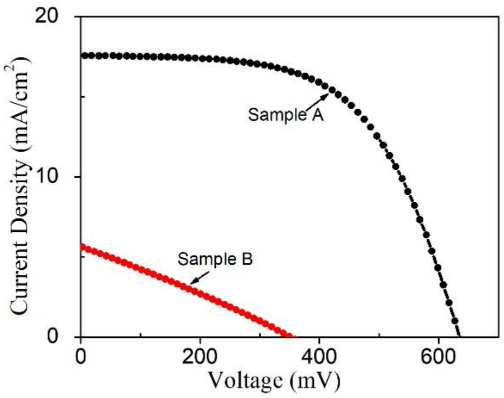

Figure S2. J-V curve of CZTS solar cells for (a) centrifugation 6000 rpm for 20 min; (b) 1500 rpm for 10 min

Table S1. solar cell performance of CZTS solar cells

Sample No.	Centrifugation condition	V_{oc} (mV)	J_{sc} (mA/cm^2)	FF (%)	E_{ff} (%)
(a)	6000 rpm for 20 min	633.3	17.6	55.8	6.2
(b)	1500 rpm for 10 min	350.2	5.6	27.4	0.54

Figure S2 J-V curves of CZTS solar cells with different centrifugation conditions. Solar cell performance was shown in Table S1. It can be concluded that centrifugation condition played very important role in both film quality and solar cell performance.
